# *Mycoplasma hominis* Causes DNA Damage and Cell Death in Primary Human Keratinocytes

**DOI:** 10.3390/microorganisms10101962

**Published:** 2022-10-01

**Authors:** Aline Teixeira Amorim, Vanesca de Souza Lino, Lucas Miranda Marques, Davi Jardim Martins, Antonio Carlos Ricardo Braga Junior, Guilherme Barreto Campos, Caline Novais Teixeira Oliveira, Enrique Boccardo, Jorge Timenetsky

**Affiliations:** 1 Department of Microbiology, Instituto de Ciências Biomédicas, Universidade de São Paulo, São Paulo 05508-900, Brazil; 2 Multidisciplinary Institute of Health, Federal University of Bahia, Vitória da Conquista 45029-094, Brazil

**Keywords:** *Mycoplasma hominis*, primary human keratinocytes, DNA damage, cell cycle, apoptosis, Toll-like receptor, oxidative stress

## Abstract

*Mycoplasma hominis* can be isolated from the human urogenital tract. However, its interaction with the host remains poorly understood. In this study, we aimed to assess the effects of *M. hominis* infection on primary human keratinocytes (PHKs). Cells were quantified at different phases of the cell cycle. Proteins involved in cell cycle regulation and apoptosis progression were evaluated. The expression of genes encoding proteins that are associated with the DNA damage response and Toll-like receptor pathways was evaluated, and the cytokines involved in inflammatory responses were quantified. A greater number of keratinocytes were observed in the Sub-G0/G1 phase after infection with *M. hominis*. In the viable keratinocytes, infection resulted in G2/M-phase arrest; GADD45A expression was increased, as was the expression of proteins such as p53, p27, and p21 and others involved in apoptosis regulation and oxidative stress. In infected PHKs, the expression of genes associated with the Toll-like receptor pathways showed a change, and the production of IFN-γ, interleukin (IL) 1β, IL-18, IL-6, and tumour necrosis factor alpha increased. The infection of PHKs by *M. hominis* causes cellular damage that can affect the cell cycle by activating the response pathways to cellular damage, oxidative stress, and Toll-like receptors. Overall, this response culminated in the reduction of cell proliferation/viability in vitro.

## 1. Introduction

*Mycoplasma hominis* has been associated with the urogenital tract microbiota. However, indirect evidence from several studies has demonstrated the pathogenic potential of this species [1,2,3]. *M. hominis* is more frequently detected in the urogenital tract of women than men and has been detected in cases of vaginitis, bacterial vaginosis (BV), or intrauterine infections [4]. After it was confirmed that *Gardnerella vaginalis* is not the only aetiological agent responsible for BV, several studies investigated the role of *M. hominis* in BV [5,6]. BV is a clinical condition characterised by the depletion of vaginal lactobacilli, pathogen colonisation, and increased bacterial load of other facultative or strictly anaerobic bacterial species [7,8]. Currently, the role of *M. hominis* in BV is accepted but considered controversial [7,9,10,11].

This pathogen has been associated with pelvic inflammatory disease and has been isolated from the amniotic fluid of women with chorioamnionitis and subsequent abortion, which indicates its association with such events [12]. The pathogen has also been associated with septicaemias and respiratory infections in patients undergoing transplants or immunocompromised individuals with joint infections. This microorganism has gained prominence in the field of medicine because it displays resistance to several classes of antibiotics, including macrolides, such as azithromycin [13]. However, the results of isolated epidemiological studies cannot be used to assess the unique characteristics of the microorganism in the establishment of infections. 

The pathogenesis of *M. hominis* infection is mediated by membrane proteins, which facilitate its adherence to host cells. In addition, damages caused by membrane enzymes, such as phospholipases and aminopeptidases, or ammonia production are potential factors that contribute to its pathogenesis [14,15]. *M. hominis* can: (a) adhere to and invade spermatozoa [16]; (b) affect immune response and signal transduction pathways; and (c) modulate the expression of genes involved in the regulation of cell growth, infection cycles, and cell death pathways in HeLa cells [1,17]. Mollicutes spp. were related to the deregulation of the cell cycle and programmed cell death of 32D murine cells [18]. The findings from some studies have shown the association of inflammation caused by chronic *M. hominis* infection with cell transformation, genomic instability, changes in p53 expression, inhibition of apoptosis, and development of prostate cancer [3,19,20,21,22,23,24]. However, these studies were conducted using immortalised and transformed cells or the tissues of patients with neoplasms; hence, the effects of infection by *M. hominis* in normal human cells, such as primary human keratinocytes (PHKs), cannot be considered to be similar to those reported in these studies. In the present study, we used commercially available primary keratinocytes, pooled from new-born foreskin, which are obtained from different healthy individuals and are commonly used as models for the study of physiology and pathology of the anogenital tract. Pooling reduces observational biases that are a consequence of a specific, homogeneous genetic background. Finally, these keratinocytes are also easier to obtain, compared with cervical or vaginal derived keratinocytes that, as an additional caveat, are usually derived from a single donor.

Owing to the uncertainties about the role of *M. hominis* in the development of pathological processes, studies that aim to improve our understanding of the alterations caused by this microorganism in the host are important—in particular, for the improvement of therapeutic measures. Therefore, the objective of the present study was to assess in vitro how *M. hominis* infection affects cell viability and induces changes in the DNA damage response pathways and inflammatory responses in PHKs.

## 2. Materials and Methods

### 2.1. M. hominis

The strain *M. hominis* (ATCC 23114-PG-21) was obtained from the ICB/USP Mycoplasma laboratory and grown in 100 mL of SP4 medium [25] until the mid-log phase. The culture was then centrifuged at 18,000× *g* for 30 min in a refrigerated centrifuge. Following this, the supernatant was removed, and the sediment was washed twice with sterile 1× phosphate-buffered saline (PBS) and recentrifuged at 18,000× *g* for 30 min at 4 °C. The sediment was then homogenised in 10 mL (10×) of the medium used to culture the PHKs without gentamicin sulphate or amphotericin. Later, the microorganisms were separated into 1-mL aliquots and stored at −80 °C until inoculation.

### 2.2. Cell Line

Low-passage (passages 1–3) PHKs derived from the foreskin of new-born (Lonza, Walkersville, MD, USA) were used. PHKs were grown in KBM Gold Basal Medium Keratinocyte Growth Medium (Lonza) supplemented with a KGM Gold Single Quots Supplement pack (Lonza) without gentamicin sulphate or amphotericin.

### 2.3. Infectious Dose and Growth Curve

To assess the effect of *M. hominis* on PHKs, infections were induced with four different doses of *M. hominis* (at 1:2, 1:10, 1:50, and 1:100 ratios) based on the stock solution prepared, as described above. For the infectious dose assay, the cells were plated at a density of 8.0 × 10^3^ to 1.5 × 10^4^ cells/mL. To observe the concentration-dependent behaviour of the cells during the 7-day infection period, a growth curve was prepared based on the initial density of 2.5 × 10^3^–8.0 × 10^3^ cells/mL. *M. hominis* with multiplicities of infection (MOIs) of 0.04–0.6 were considered for the replicates. All cells were grown in 24-well cell culture microplates (Costar) for 12–24 h to facilitate adherence to the well before infection. The culture medium was refreshed every 2 days without microorganisms [26,27]. For preparing the growth curve and determining the infectious dose, the cells were washed with 1× PBS, harvested with 0.5% Trypsin-EDTA 1× (Gibco), resuspended in D10 medium, and enumerated using a haemocytometer. For determining the infectious dose, the cells were quantified after 72 h of infection. To obtain the growth curve, the cells were counted each day for 7 consecutive days.

### 2.4. Clonogenic Assay

A clonogenic assay was performed to test the survival and proliferation of cells after infection with microorganisms at different concentrations [28]. One hundred PHKs were plated in KBM Gold Basal Medium Keratinocyte Growth Medium (Lonza) supplemented with a KGM Gold Single Quots Supplement pack (Lonza) without gentamicin sulphate or amphotericin at 37 °C and 5% CO_2_. After 12 h, the cells were inoculated with *M. hominis* (MOI, 1:30) and monitored for 15 days. The culture medium was refreshed every 2 days by adding fresh culture medium without microorganisms. After 15 days, the cells were washed with 1× PBS, fixed with 10% formaldehyde (Merck Millipore, Darmstadt, Germany), and stained with 1 mL of crystal violet solution (0.1% crystal violet and 2% absolute ethanol). After staining, the plates were washed twice with water and imaged, and the number of colonies was counted.

### 2.5. Sub-G0/G1 DNA Content and Cell Cycle Analyses Using Flow Cytometry

The DNA content in cells from the sub-G0/G1 phase and changes in the cell populations in the different phases of the cell cycle were assessed in PHKs infected with *M. hominis*. The cells were stained with propidium iodide (PI) and analysed by flow cytometry in accordance with a modified version of a protocol reported by Quinet et al. (2014) [29]. Accordingly, 150,000 PHKs were infected with *M. hominis* and incubated at 37 °C for 6 and 48 h. After this period, the cells were trypsinised and centrifuged along with their supernatants at 1500–2000× *g* for 5 min. The cells were fixed with 70% frozen ethanol and stored at −20 °C for at least 24 h. The samples were washed with 1× PBS and centrifuged (1500× *g*, 5 min), and the pellet (with the DNA) was resuspended in PI solution (20 μg/mL PI, 200 μg/mL RNase A, and 0.1% Triton X-100) and incubated for 30 min in the dark at room temperature. The samples were rewashed with 1× PBS, centrifuged (4000× *g*, 3 min), and homogenised with 100–200 μL of filtered 1× PBS, depending on the size of the pellet. Following this, the samples were transferred to microtubes, and PI fluorescence was evaluated using flow cytometry (BD Accuri C6 Cytometer). Approximately 10,000–25,000 cells were analysed for each sample, and the acquisition rate was 100 events/second for the differentiation of ‘singlets’ and ‘doublets’. In addition, an FSC vs. PI fluorescence cytogram was prepared to exclude the signals from cellular debris. The data were analysed using the BD Accuri C6 software. The histograms of fluorescence emitted were analysed to detect and quantify the cells with fragmented DNA (sub-G0/G1 phase), normal DNA (diploid, G0/G1 phase, before DNA synthesis), DNA undergoing replication (S phase), and duplicated DNA (polyploid, G2/M phase).

### 2.6. Protein Extraction and Immunoblotting

After 48 h of *M. hominis* infection, the PHKs were lysed to isolate the proteins. Initially, the culture supernatant was removed, and 400–1000 μL of lysis buffer provided in the Human Apoptosis Array kit (Proteome Profiler, R&D Systems, Minneapolis, MN, USA) supplemented with protease inhibitors (Complete Mini, Roche Diagnostics, GmbH, Mannheim, Germany) was added to the cell monolayer. For immunoblotting, 400–1000 μL of RIPA lysis buffer (150 mM NaCl, 5 mM EDTA, 50 mM Tris-HCl (pH 8.0), 0.1% NP-40, 0.5% Na-deoxycholate, and 0.1% sodium dodecyl sulphate (SDS)) supplemented with protease inhibitors (Complete Mini, Roche Diagnostics) was added to the monolayer. The protein concentration in the samples used for the Human Apoptosis Array was measured using the Pierce BCA Protein Assay kit (Thermo Fisher Scientific, Waltham, MA, USA), and the Bio-Rad protein assay kit (Bio-Rad, Hercules, CA, USA) was used for immunoblotting the samples.

Proteins involved in the regulation/activation of PHK apoptosis were evaluated using the Human Apoptosis Array (Proteome Profiler, R&D Systems, Minneapolis, MN, USA). After the protein extracts were quantified, the samples were diluted to 200 μg, distributed in the kit membrane, and incubated under agitation for 24 h at 4 °C according to the manufacturer’s instructions. The protein bands were analysed using the image quantification software ImageJ (http://imagej.nih.gov/ij/; accessed on 16 March 2021).

For protein analysis by immunoblotting, 30 μg of the total protein extract was denatured and separated by SDS-polyacrylamide gel electrophoresis and transferred to a polyvinylidene fluoride membrane. Anti-PCNA (1:500; Life Technologies, Carlsbad, CA, USA, 180110), anti-p27 (Novocastra, 117708, São Paulo, Brazil, 01156-060), anti-cyclin A (Novocastra, 117209), anti-p53 (1:500; Sigma, P6874), anti-cyclin E (1:500; BD Pharmingen, San Diego, CA, USA, 551170), and anti-tubulin (1:10,000; Sigma, St. Louis, MO, USA; T9026) antibodies were used as the primary antibodies. Anti-mouse (1:1000; 115-035-003) or anti-rabbit (1:1000; 111-035-003) antibodies conjugated with horseradish peroxidase (Jackson ImmunoResearch, West Grove, PA, USA) were used as the secondary antibodies. The antibodies were prepared according to the manufacturers’ instructions. The peroxidase activity of the secondary antibodies was assessed using an enhanced chemiluminescence kit (Amersham Biosciences, GE Healthcare, Chicago, IL, USA). The signals were quantified using the ImageJ software.

### 2.7. Cytokine Quantification 

Cytokines released in the cell culture supernatant were quantified using the Human Apoptosis Array. The commercial kit ProcartaPlex^®^ Immunoassay Th1/Th2/Th9/Th17/Th22/Tfh/Treg (18 plex) (eBioscience, Affymetrix, USA) was used to determine the levels of GM-CSF, IFN-γ, interleukin (IL) 1β, IL-2, IL-4, IL-5, IL-6, IL-9, IL-10, IL-12p70, IL-13, IL-17A, IL-18, IL-21, IL-22, IL-23, IL-27, and tumour necrosis factor alpha (TNF-α). The cytokine levels were measured according to the manufacturer’s instructions and normalised to the level of total proteins obtained from 1000-μg cell extracts.

### 2.8. RNA Extraction and Real-Time Quantitative Polymerase Chain Reaction (RT-qPCR)

For RT-qPCR assays of genes related to the Toll-like receptor and DNA damage response pathways, *M. hominis* infection was performed for 1 and 48 h in accordance with a modified version of the protocol reported by Hopfe et al. (2013) [1]. Total RNA was extracted by the direct application of TRIzol (Invitrogen) and purified with chloroform and isopropanol. At the end of the extraction process, the samples were treated with RQ1 RNase-free DNAse (Promega, Madison, WI, USA), and their optical density was measured at 260 nm. cDNA was prepared with 0.5 μg of RNA from each sample using the RT2 First Strand kit (Qiagen, Hilden, Germany) in accordance with the manufacturer’s instructions. Finally, the cDNA obtained was subjected to a gene expression analysis using the Human Toll-Like Receptor Signalling Pathway and Human DNA Damage Signalling Pathway kits (Qiagen-SABioscience, São Paulo, Brazil), according to the manufacturer’s instructions. Amplifications were performed using the StepOnePlus Real-Time PCR System (Applied Biosystems). Analysis was performed using the RT² Profiler PCR Array Data Analysis online software (Qiagen).

### 2.9. Statistical Analysis

The Mann–Whitney one-tailed nonparametric test was used for all assays when the analyses were performed between two independent groups, since the prerequisites for a parametric test were not met. For cases in which the analysis involved a quantitative variable and two nominal variables, two-way analysis of variance (ANOVA) was performed with the Bonferroni post-test. For all tests, statistical significance was set at *p* < 0.05 with a 95% confidence level.

Data were analysed using Prism v5 software (GraphPad Software, San Diego, CA, USA) with the exception of the gene expression data, which were generated and analysed using the kit software (https://dataanalysis2.qiagen.com/pcr; accessed on 18 February 2020). GraphPad software was used for the graphical presentation of the results.

## 3. Results

### 3.1. M. hominis Infection Alters the Growth of Keratinocytes Even at Low Doses

**Figure 1:**
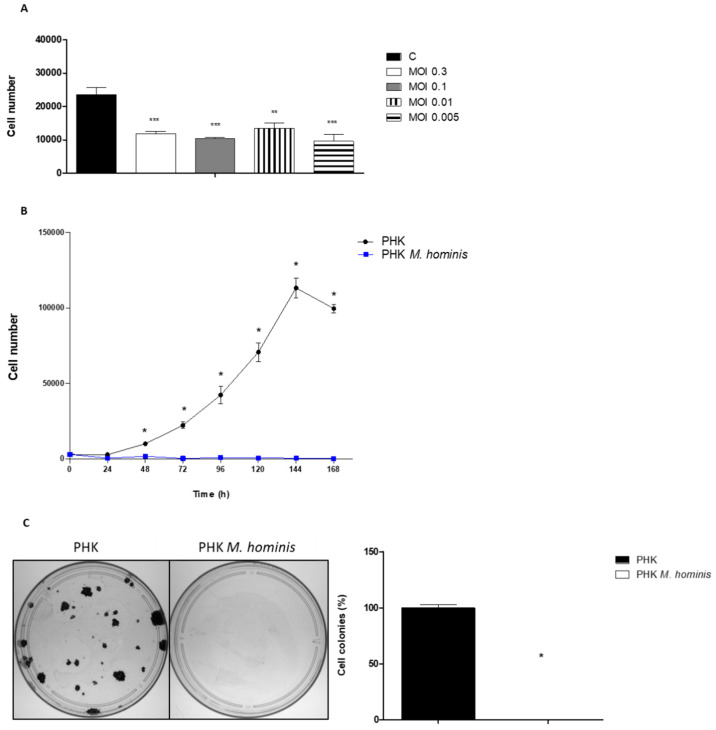
*Mycoplasma hominis* infection alters the growth and colony formation of primary human keratinocytes (PHKs) even at low inoculum concentrations. (**A**) Quantification of viable PHKs after 72 h of infection with *M. hominis* at different concentrations. Approximately 8000 PHKs/well were present at the time of infection. * Represents a statistically significant difference between the infection and control groups (one-tailed Mann–Whitney test, significance level of 95%, (*) *p* ≥ 0.05, (**) *p* ≥ 0.01, and (***) *p* ≥ 0.001). (**B**) PHK growth curves after *M. hominis* infection. Plating was performed with approximately 2.3 × 10^3^ to 3.7 × 10^3^ cells. After 12–24 h, *M. hominis* infection was induced at a multiplicity of infection (MOI) of 0.04–0.6. After 24, 48, 72, 96, 120,144, and 168 h of infection, the cells were trypsinised and counted using a haemocytometer. The assays were performed in triplicate. The points on the curves represent the average number of cells obtained with the respective standard deviations. From 48 h of *M. hominis* infection, a statistically significant difference was observed in the infection group compared with that in the control group (two-way ANOVA with Bonferroni post-test, significance level of 95%). (**C**) The clonogenic assay performed with PHKs after *M. hominis* infection. The number of PHKs initially plated was approximately 1000. The MOI for *M. hominis* was 4.44. Left: photograph of the petri dishes in one of the tests. The colonies were stained with crystal violet and counted using the Promega Colony Counter (Promega). Right: reduction in the colony formation percentage after 15 days of infection. One-tailed Mann–Whitney test, significance level of 95%.

To assess whether *M. hominis* could alter cell growth, we performed an infectious dose assay. The findings confirmed that *M. hominis* infection decreased PHK viability even at low inoculum concentrations (Figure 1A). Since *M. hominis* caused changes in keratinocyte growth at all inoculum concentrations, an MOI of 0.04–0.6 was used for the cell growth curve assays. The proliferation of cells infected with *M. hominis* showed an overall decline, with a statistically significant difference observed from 48 h of infection (Figure 1B). After this period, cell growth was not restored. A clonogenic assay was performed to test the survival and proliferative capacity of the cells after 15 days of infection without the replacement of the pathogen. *M. hominis*-infected keratinocytes did not show colony formation after the infection period (Figure 1C). Therefore, *M. hominis* prevented the growth of keratinocytes early after infection (48 h). 

### 3.2. Infection by M. hominis Alters the Cell Cycle, Thus Promoting the Activation of DNA Damage and Oxidative Stress Signaling Pathways in Keratinocytes

**Figure 2:**
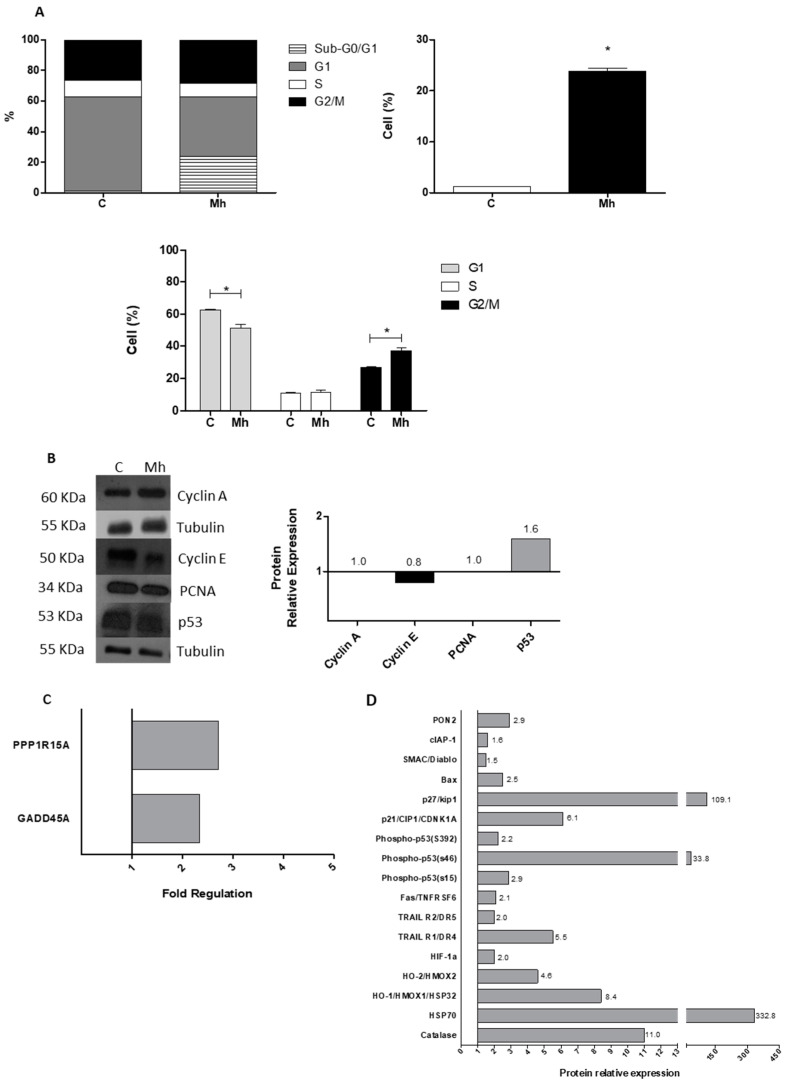
*M. hominis* infection affects the progression of PHK cell cycle and induces DNA damage by altering the expression of proteins related to apoptosis regulation. (**A**) Analysis of PHKs at different phases of the cell cycle after *M. hominis* (Mh) infection. Analysis was performed using data obtained from flow cytometry histograms prepared after *M. hominis* infection using MOI 0.01–0.04 in 1.5 × 10^5^ PHKs. The cells were collected after 48 h of infection. Left panel: stacked column chart representing the percentage of cells in the Sub-G0/G1, G1, S, and G2/M phases of the cell cycle. Right panel: percentage of cells in the Sub-G0/G1 phase. Statistical significance (*p* < 0.05) is indicated by an asterisk (*) (Mann–Whitney, one-tailed test). Lower panel: The bars represent the average percentage of viable cells in the G1, S, and G2/M phases. The brackets represent the groups that showed statistically significant differences (Mann–Whitney, one-tailed test, *p* ≤ 0.001). (**B**) Expression of proteins involved in cell cycle regulation. The levels of cyclin A, cyclin E, PCNA, and p53 were determined by Western blotting. Total protein extracts (30 μg) obtained from *M. hominis*-infected and control PHKs. The proteins were separated in polyacrylamide gel; transferred to a PVDF membrane; and analysed in the presence of anti-cyclin A, anti-cyclin E, anti-PCNA, and anti-p53 antibodies. The reaction was evaluated using the chemiluminescence method, and the bands were quantified using ImageJ. Right panel: relative expression of proteins from Mh-infected cells compared with tubulin expression and control cells. (**C**) Expression analysis of genes associated with the DNA damage pathways in *M. hominis*-infected PHKs after 48 h. The genes shown exhibited statistically significant differences in expression (Mann–Whitney non-parametric test, *p* < 0.05, one-tailed test, significance level: 95%). (**D**) Relative expression of proteins involved in the regulation/mediation of apoptosis. Expression was evaluated in 200 μg of total protein extracts obtained from monolayered *M. hominis*-infected and control PHKs. The assay was performed using the Human Apoptosis Array (R&D Systems). The results were analysed using ImageJ software and tabulated using GraphPad Prism. The assay was performed in duplicate. The bars represent the relative protein expression. Only proteins that showed statistically significant differences in expression between the control and infected groups in the Student’s *t*-test are shown; significance level: 95%.

To evaluate whether the distribution of keratinocytes in the different phases of the cell cycle was altered upon *M. hominis* infection, the cells in phases G1, S, and G2/M of the cell cycle were quantified by flow cytometry. Concurrently, the number of cells present in the sub-G0/G1 phase was analysed to confirm whether *M. hominis* infection could cause cell damage and thus lead to DNA fragmentation, which may or may not have induced apoptosis in the initial stage of the infection. A clear increase in the percentage of keratinocytes in the sub-G0/G1 phase was observed compared with that in the control group briefly after 6 h of *M. hominis* infection (Supplementary Figure S1), and the same was also observed after 48 h of infection (Figure 2A). After 6 h of infection by *M. hominis*, the number of cells in the S phase increased, and the number of cells in the G2/M phase decreased (Supplementary Figure S1). After 48 h of infection, a decline in the G1 population was observed, whereas the frequency of cells in the G2/M phase increased (Figure 2A). Hence, *M. hominis* infection increased the number of PHKs in the sub-G0/G1 and S phases after 6 h of infection and that in the G2/M phase after 48 h of infection.

To verify whether *M. hominis* infection could alter the expression of proteins involved in the cell cycle, the total protein extracts were isolated from infected cells after 48 h of infection and analysed by Western blotting. No changes were observed in the expression of cyclin A and PCNA (Figure 2B), whereas the expression of cyclin E was lowered in *M. hominis*-infected cells. 

To determine how p53 expression was increased (Figure 2B,D), the expression of genes encoding proteins related to the DNA damage pathways was analysed in PHKs after 48 h of *M. hominis* infection for confirming the involvement of the DNA damage pathways. Only genes that showed more than two-fold difference in expression compared with the control group were considered for statistical analyses. Figure 2C shows that, in PHKs, only two genes were upregulated (*GADD45A* and *PPP1R15A*) after 48 h of infection. *M. hominis* infection altered the expression profile of some genes related to DNA damage pathways in PHKs, thus promoting p53 expression. 

Given that the number of cells in the dose-dependent assay, growth curve, and clonogenic assay decreased, the number of cells in the sub-G0/G1 phase increased, and the expression of some specific DNA damage response genes was altered, and changes in the expression of 35 proteins involved in the regulation/mediation of apoptosis were evaluated using the Human Apoptosis Array kit. Figure 2D shows the results of the analysis of the proteins detected. A difference was observed in the expression of 17 pro- and antiapoptotic proteins in PHKs after 48 h of *M. hominis* infection. Overall, the expression of Pon2; cIAP-1; SMAC-Diablo; Bax; p27/kip1; p21; p53 (phosphorylated at the serine residues at positions 15, 46, and 392); Fas/TNFRSF6; Trail R1 and R2; HIF-IA; HO-1; HO -2; HPS 70; and catalase increased. In summary, upregulation of the proapoptotic factors was predominant. Therefore, *M. hominis* can alter protein expression in the pathways involved in the regulation of PHK apoptosis, primarily those involved in the oxidative stress response.

### 3.3. M. hominis Infection Alters the Expression of Toll-like Receptors and Induces the Release of Pro-Inflammatory Cytokines

**Figure 3:**
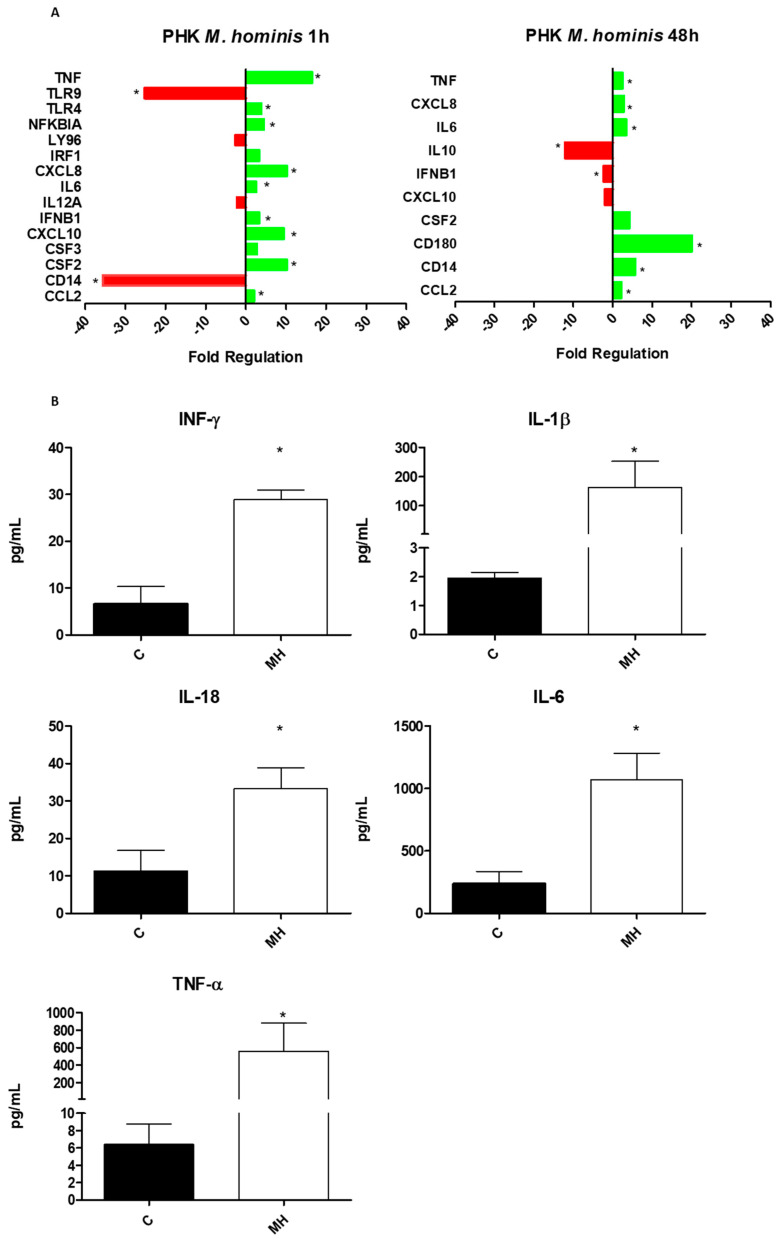
*M. hominis* alters the expression of genes encoding proteins related to the Toll-like receptor pathways, leading to the release of proinflammatory cytokines by PHKs. (**A**) Expression analysis of the genes encoding proteins related to the Toll-like receptor pathways in PHKs infected with *M. hominis* after 1 (left) and 48 h (right) of infection. Positive regulation (green) and negative regulation (red) of genes associated with the activation of innate and acquired immune responses. Statistical significance (*p* < 0.05) is indicated by an asterisk (*) (Mann–Whitney non-parametric test, one-tailed test). (**B**) Quantification of cytokines released in the PHK culture supernatant after 48 h of *M. hominis* infection. The IFN-γ, IL-1β, IL-18, IL-6, and TNF-α levels were normalised to the total protein expression in 1000-μg cell extracts. Data are expressed as the median ± standard deviation. * Represent a statistically significant difference between the infected and control groups (Mann–Whitney one-tailed, *p* ≤ 0.05).

The expression of genes encoding proteins involved in the Toll-like receptor pathway was evaluated in keratinocytes after 1 and 48 h of *M. hominis* infection (Figure 3A). For statistical analyses, only genes that showed a two-fold differential expression in infected cells vs. uninfected cells (control) were considered. After 1 h of infection, *CCL2, CSF2, CXCL10, IFNB1, IL6, CXCL8, NFKBIA, TLR4*, and *TNF* were upregulated, whereas *CD14*, *IL12A*, *LY96*, and *TLR9* were downregulated (Figure 3A, left panel). After 48 h, *CCL2*, *CD14*, *CD180*, *IL6*, and *TNF* were upregulated, whereas *IFNB1* and *IL10* were downregulated (Figure 3A, right panel). Changes in the expression of multiple genes, such as *CD14*, *INFB1*, and *CXCL10*, varied with the infection duration. Hence, *M. hominis* infection can alter the expression of genes encoding proteins related to the Toll-like receptor pathways in PHKs in a manner that varies with the duration of infection. Moreover, the infected PHKs released IFN-γ, IL-1β, IL-18, IL-6, and TNF-α at higher levels, exhibiting an overall proinflammatory profile (Figure 3B).

## 4. Discussion

In this study, we demonstrated that infection with *M. hominis* at different inoculum concentrations exerted a significant effect on the number of keratinocytes. We observed a considerable decrease in the number of viable cells during 7 days of infection, which indicates that *M. hominis* alters the proliferation and/or viability of PHKs. These effects were also reflected in the clonogenic potential of these cells, as PHK colonies were found to be absent after 15 days of infection. These data are important, because they indicate the deleterious effect of *M. hominis* on PHKs even at low concentrations and its ability to affect PHK growth and/or viability.

The determination of the growth curve of cell lines after experimental infection by Mollicutes and the dose-dependent effects of the microorganisms have rarely been studied. Few studies have focused on the importance of verifying the load of Mollicutes in pathological processes [30]. Often, the mere presence of a microorganism may not be directly related to the development of a disease but could lead to an increased load, because the urogenital microbiota is a complex niche; this may influence the pathogenic role of *M. hominis* [31]. Several studies have reported that women with BV show the presence of *M. hominis* more often and at higher loads than women without BV [7,32,33]. Therefore, the detection and quantification of Mollicutes can be used to monitor and treat patients with antibiotics [31,34]. The findings of this study highlight the importance of quantifying *M. hominis* during routine diagnostic tests, since not only their presence but also their load may influence female sexual health.

*M. hominis* affects the PHK cell cycle, as indicated by the increased abundance of cells in the sub-G0/G1 phase. This is an indication of DNA fragmentation, which is often related to apoptosis, after 6 and 48 h of infection. In addition, infection increased the residual cells in the S and G2/M phases after 6 and 48 h of infection, respectively. After 48 h, we observed an increase in the expression of GADD45 and PPP1R15A. In addition to this, high levels of p21 and p53 phosphorylation were observed in the infected cells. GADD45 transcription can be induced by increasing the phosphorylation of p53 at the serine residue at position 15, which occurs during DNA damage [35]. Gadd45 proteins were observed to interact with p21, a p53 target protein that is a universal cyclin-dependent kinase inhibitor implicated in G1/S and G2/M cell cycle arrest [36,37,38]. 

When the expression of the proteins involved in the cell cycle or DNA replication was evaluated, no difference was observed in the expression of cyclin A and PCNA. However, the expression of cyclin E decreased. This could be explained by the increased expression of p27 (kip 1) (Figure 2D), which binds to and inhibits the expression of cyclin E/CDK2 [39]. 

PPP1R15A (also known as GADD34) is also involved in the response to DNA damage [40,41]. These findings suggest that *M. hominis* infection caused cell damage and eventually induced cell cycle arrest; however, the cell fate depends on the extent of damage. The response to genotoxic stress in mammalian cells is complex and can involve several processes. Transient cell cycle arrest prevents the replication of damaged DNA, which allows p53 to facilitate the transcription and translation of proteins involved in DNA repair. If repair is not possible, pathways such as apoptosis, which lead to programmed cell death, can be activated to prevent the replication of cells with abnormal DNA [42,43]. As p53 plays a key role in regulating these pathways, the expression of p53 and its upstream and downstream proteins, such as MdM2, p21, Gadd45, Bax, and PUMA, should be tightly regulated [44]. Further, while the *TP53* (tumour protein p53) gene expression level did not change after *M. hominis* infection, the protein level of p53 increased. P53 protein stabilisation, without an increase in the transcription levels of its coding mRNA, is a common response related to DNA damage. Here, we showed that *M. hominis* infection altered the expression profile of some genes related to DNA damage pathways in PHKs, suggesting the activation of DNA damage pathways. Therefore, we can assume that in our model, p53 protein accumulation, in the absence of increased mRNA levels, can be explained, at least in part, by protein stabilisation. The molecular mechanisms involved are being investigated in our laboratory [45,46,47,48].

To better understand the mechanisms involved in cell death, the expression of 35 proteins involved in apoptosis regulation was evaluated. The expression of both pro- and antiapoptotic proteins increased. However, there was a predominance of the expression of proapoptotic proteins related to the extrinsic apoptotic pathway, such as TRAIL and Fas, and intrinsic pathway proteins, such as Bax and Smac/DIABLO [49], which explains the cell death data obtained from the growth curve and the clonogenic and sub-G0/G1-phase assays. 

Of note, *M. hominis* uses arginine to produce ATP, and arginine degradation could contribute to the apoptosis of infected cells. Occasionally, the depletion of a specific nutrient, such as choline, can play an important role in inducing apoptosis in infected cells [50]. Arginine depletion may lead to the inhibition of the mTOR pathway, thus indirectly activating DNA damage pathways, such as ATM/ATR, which may also explain the cell cycle arrest and apoptosis in cells with a high microorganism load [51]. Furthermore, mycoplasma deaminases hydrolyse L-arginine into L-citrulline and NH_3_. The latter is immediately converted into ammonia, which increases the pH of the medium. Ammonia production and an increased pH can be harmful to cells. Significant changes in the pH of the culture medium not only induce cell growth but also exert toxic effects on cells, thus causing programmed cell death [52,53]. 

Catalase expression was found to increase in the PHKs, similar to other proteins involved in the oxidative stress response, such as Ho-1, Ho-2, HIF-1α, and HSP70 [54,55,56], which may indicate that the cells attempted to combat oxidative stress induced by *M. hominis*. Chen et al. (2005) reported that the level of H_2_O_2_ in vaginal discharge collected from patients with BV was approximately ten times higher than that in vaginal discharge collected from healthy women, which indicates oxidative stress in the vaginal environment in the former case [57]. Mollicutes produce H_2_O_2_ and other products related to oxidative stress [58,59,60,61,62,63]. As *M. hominis* produces ammonia as a metabolic product, oxidative stress is well-known to be strongly associated with ammonia toxicity and is characterised by excess ROS/RNS production or impairment of its detoxification via endogenous mechanisms. In this condition, the oxidation of lipids and proteins may occur, causing cellular damage to some cell components, DNA damage, impairment of cellular function, and induction of cell death [64,65,66,67,68,69]. Thus, studies on the role of *M. hominis* in triggering oxidative stress and inducing apoptosis are important to understand its role in pathological processes. Additionally, the substantial expression of HSP70 can be explained by the inflammatory environment triggered in response to infection (through components such as TNF-α and IL-1), since HPS70 can provide protection from toxicity and cell death mediated by reactive oxygen species (including NO) and cytokines (TNF-α and IL-1 in particular) [70,71,72].

To understand this phenomenon and the immune response profile of the cell line against *M. hominis* infection, the expression of genes encoding proteins related to the Toll-like receptor pathways, as well as the cytokines released were quantified. The expression profiles in PHKs obtained after 1 and 48 h of *M. hominis* infection were different. Differences were also observed in the release of proinflammatory cytokines. These differences may be associated with the adaptive process of the pathogen to the cells. After 1 h of infection by *M. hominis*, an increase in *TNF*, *TLR4*, *NFKBIA*, *CXCL8*, *IL6*, *IFNB1*, *CXCL10*, *CSF2*, and *CCL2* levels and a decrease in *TLR9* and *CD14* levels were observed. After 48 h of infection, the levels of *TNF*, *CXCL8*, *IL6*, *CSF2*, *CD180*, *CD14*, and *CCL2* levels increased and those of *IL-10*, *INFB1*, and *CXCL10* decreased. An increase in the levels of the cytokines IL-1B, IL-18, IL-6, and TNF-α was observed after 48 h. Thus, the predominance of an inflammatory response against bacterial infection was observed, which was accentuated and established after 48 h. At the beginning of the infection (1 h), despite the increase in TLR4 expression, there was a decrease in the expression of CD14 and LY96 (also known as MD-2), which are important for lipopolysaccharide recognition [73]. This molecular profile is important for the onset of the innate immune response, which indicates the adaptation of the cell response to the microorganism. Bacterial pathogens can induce the release of pro-inflammatory cytokines, such as TNF-α, IL-6, and IL-12, by stimulating TLR4 in the epithelial cells of the endocervix [74]. TLR4 signalling, following Mollicutes infections, is poorly described. Ureaplasmal or mycoplasmal LAMPs have been shown to interact with TLR2 and TLR4, leading to an inflammatory response [75,76]. Mycoplasma species do not contain common ligands for TLR4, such as lipopolysaccharides, peptidoglycans, and lipoteichoic acid, but contain an abundance of acylated lipoproteins, such as cell surface antigens, which are recognised as pathogen-associated molecular patterns. As TLR4 may recognise components other than lipopolysaccharides, studies should be performed to verify this association for *M. hominis* [77]. In a study conducted by Abrahams et al. (2013), *M. hominis* increased the mRNA expression of TLR4, TLR6, and TLR8 in cells from the human foetal membrane [78]. The increased expression of TLR4 after Mollicutes infections may be associated with the induction of autophagy in the cells [77]. Another mechanism for the induction of apoptosis by mycoplasma is the increase in apoptotic signalling through specific interaction of mycoplasmas with cell surface receptors or alteration of apoptosis signalling pathways [3].

In addition, in the present study, *M. hominis* was found to reduce TLR9 expression. Infection by another species of Mollicutes, *M. pulmonis*, was previously shown to interfere with the endosomal system of the host; it disrupted the dynamics of endosomes and autophagosomes by upregulating Rab7 and suppressing autophagolysosomal degradation, thereby promoting the establishment of an intracellular niche that can be exploited by mycoplasma or other intracellular pathogens [79,80]. Rab7 belongs to the family of small guanosine triphosphatases (small GTPases) and is localised to certain organelles. Rab7 participates in the nutrition and apoptosis of cells under regulation by growth factors, mediating the internalisation and degradation of nutrient transporters. Thus, the expression of this gene, which could be enhanced under mycoplasma infection, was also related to the suppression of TLR9 [81]. This would also explain the decrease in the gene expression of IFN-β after 48 h of infection. However, as the increased production of cytokines and chemokines associated with the inflammatory process was predominant after 48 h of infection, the potential involvement of alternative activation pathways, such as CD180 (RP105), in Mollicutes is worth investigating, even though the role of CD180 in regulating the inflammatory response is cell type-dependent, as observed in mycobacterial species [82]. As the role of *M. hominis* in the modulation of Rab7, TLR9, and CD180 has not been reported, additional studies are necessary to elucidate the *M. hominis*–PHK interaction in the infection process.

## 5. Conclusions

This is the first study to assess the effect of *M. hominis* infection on PHKs harvested from the foreskin. *M. hominis* infection induced changes in both the proliferation and viability of keratinocytes. The cell damage affected the cell cycle, activated the cell damage response pathways, and altered the expression of proteins involved in the regulation of apoptosis and oxidative stress and genes encoding Toll-like receptors, thus inducing a proinflammatory response. These data are relevant and provide a broad overview of the role of *M. hominis* in the genital microenvironment and may contribute to the development of effective therapeutic measures to control infections caused by this type of Mollicutes.

## Data Availability

Not applicable.

## Supplementary Materials

The following supporting information can be downloaded at https://www.mdpi.com/article/10.3390/microorganisms10101962/s1: Figure S1: *Mycoplasma hominis* infection affects the progression of the PHK cell cycle after 6 h of infection.
