# Supplementary material for: Mycoplasma hominis Causes DNA Damage and Cell Death in Primary Human Keratinocytes
Source: Microorganisms. 2022 Oct 1;10(10):1962. doi: 10.3390/microorganisms10101962 (PMC9608843; doi:10.3390/microorganisms10101962)
Supplement: Supplementary file 1 [file microorganisms-10-01962-s001.zip › microorganisms-1884078-supplementary.pdf]

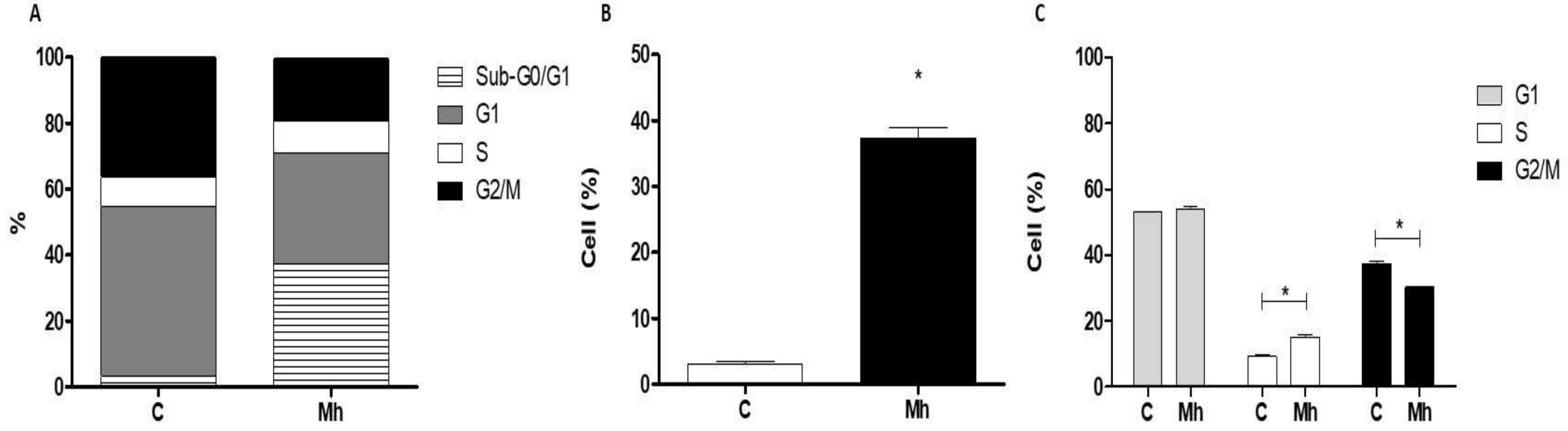


Figure S1: *Mycoplasma hominis* infection affects the progression of the PHK cell cycle after 6 h of infection. Statistical significance ($p < 0.05$) is indicated by an asterisk.
